# An Improved Teaching-Learning-Based Optimization Algorithm with Reinforcement Learning Strategy for Solving Optimization Problems

**DOI:** 10.1155/2022/1535957

**Published:** 2022-03-24

**Authors:** Di Wu, Shuang Wang, Qingxin Liu, Laith Abualigah, Heming Jia

**Affiliations:** ^1^School of Education and Music, Sanming University, Sanming 365004, China; ^2^School of Information Engineering, Sanming University, Sanming 365004, China; ^3^School of Computer Science and Technology, Hainan University, Haikou 570228, China; ^4^Research and Innovation Department, Skyline University College, Sharjah 1797, UAE; ^5^Faculty of Computer Sciences and Informatics, Amman Arab University, Amman 11953, Jordan; ^6^School of Computer Science, Universiti Sains Malaysia, George Town, Pulau Pinang 11800, Malaysia

## Abstract

This paper presents an improved teaching-learning-based optimization (TLBO) algorithm for solving optimization problems, called RLTLBO. First, a new learning mode considering the effect of the teacher is presented. Second, the Q-Learning method in reinforcement learning (RL) is introduced to build a switching mechanism between two different learning modes in the learner phase. Finally, ROBL is adopted after both the teacher and learner phases to improve the local optima avoidance ability of RLTLBO. These two strategies effectively enhance the convergence speed and accuracy of the proposed algorithm. RLTLBO is analyzed on 23 standard benchmark functions and eight CEC2017 test functions to verify the optimization performance. The results reveal that proposed algorithm provides effective and efficient performance in solving benchmark test functions. Moreover, RLTLBO is also applied to solve eight industrial engineering design problems. Compared with the basic TLBO and seven state-of-the-art algorithms, the results illustrate that RLTLBO has superior performance and promising prospects for dealing with real-world optimization problems. The source codes of the RLTLBO are publicly available at https://github.com/WangShuang92/RLTLBO.

## 1. Introduction

In recent years, real-world optimization problems have become increasingly complex and diverse in a wide range of fields and disciplines. Traditional (mathematical) optimization methods, such as Newton's method and the gradient descent method can no longer meet the needs for solving current optimization problems. Thus, nontraditional methods, especially metaheuristic algorithms, are becoming increasingly pervasive among researchers [1–3]. Metaheuristics are algorithms based on intuition or experience, that can provide a feasible solution at an acceptable cost (referring to computing time and computational resources), and the deviation between the feasible solution and the optimal solution may not be predicted in advance. Metaheuristic optimization algorithms have the merits of being flexible, having few parameters and avoiding local optima. Additionally, they can be rapidly deployed and thus have been utilized for solving various optimization problems over the past decades [4, 5]. Some of the most representative meta-heuristic algorithms are listed as follows: genetic algorithms (GA) [6], differential evolution algorithm (DE) [7], simulated annealing (SA) [8], arithmetic optimization algorithm (AOA) [9], heat transfer relation-based optimization algorithm (HTOA) [10], particle swarm optimization (PSO) [11], salp swarm algorithm (SSA) [12], grey wolf optimizer (GWO) [13], whale optimization algorithm (WOA) [14], aquila optimizer (AO) [15], remora optimization algorithm (ROA) [16], etc.

Teaching-learning-based optimization (TLBO) is a meta-heuristic algorithm proposed by Rao et al. in 2011 [17]. The TLBO method is inspired by the teaching-learning process in a class and simulates the influence of a teacher on learners. Due to the advantages of rapid convergence, absence of algorithm-specific parameters and easy implementation, TLBO has become a viral optimization algorithm and has been successfully applied to real-world problems in diverse fields. Aouf et al. [18] applied TLBO to optimize the parameters of the ANFIS structure to obtain the optimal trajectory and traveling time to address the navigation problem of the mobile robot in a strange environment. Singh et al. [19] studied the application of TLBO for optimal coordination of directional overcurrent relays (DOCRs) in a looped power system. Multiobjective TLBO was applied to solve the motif discovery problem (MDP) in the bioinformatics field by Gonzalez-Alvarez et al. [20], and obtained better solutions than other biology-based multiobjective evolutionary algorithms. All the above applications have suggested that TLBO can be effectively applied to many optimization problems in various fields.

The improvement and hybrid algorithms of TLBO and their applications have also been studied by several researchers [21]. Kumar and Singh [22] developed a chaotic version of TLBO with different chaotic mechanisms. A local search method was also incorporated to guide the search direction between local and global search and to improve the quality of solution. The application of clustering problems proved the effectiveness of this algorithm. Taheri et al. [23] proposed a balanced TLBO with three modifications, called BTLBO. A weighted mean replaced the mean value in the teacher phase to maintain the diversity. The tutoring phase was added as a powerful local search mechanism for exploiting regions around the best solution. The restarting phase was introduced to improve the exploration ability by replacing inactive learners with randomly initialized learners. Ma et al. [24] proposed a modified TLBO (MTLBO) by introducing a population group mechanism into the basic TLBO. All students were divided into two groups and updated by different updating strategies. The MTLBO was also applied to establish the NOx emission model of a circulation fluidized bed boiler. Xu et al. [25] introduced dynamic-opposite learning (DOL) strategy into TLBO to overcome premature convergence. The asymmetric search space and the dynamic change in the characteristics of DOL help DOLTLBO to holistically improve the exploitation and exploration capabilities. Dong et al. [26] presented a KTLBO algorithm to achieve computationally expensive constrained optimization. The kriging-assisted two-phase optimization framework was used to alternately conduct global and local searches, achieving the search acceleration. KTLBO was also adopted to design the structure of a blended-wing-body underwater glider. Ren et al. [27] developed a multiobjective elitist feedback TLBO (MEFTO) for multiobjective optimization problems. The elitism strategy was used to store the best solutions obtained thus far. The proposed feedback phase allowed students to choose whether to study directly with the teacher or to motivate themselves, providing a novel way for students to improve themselves. Zhang et al. [28] proposed a hybrid algorithm based on TLBO and a neural network algorithm (NNA) named TLNNA to solve engineering optimization problems. The experimental results suggested that TLNNA has improved global search ability and fast convergence speed. By considering the features of the WOA and TLBO, Lakshmi and Mohanaiah [29] proposed a hybrid WOA-TLBO algorithm. This was also applied to solve the facial emotion recognition (FER) functional problem, and the reported results showed its effectiveness and high accuracy.

The TLBO variants proposed previously have improved searchability and accelerated the convergence process, but they still struggle with premature convergence and insufficient learning processes. Thus, in this paper we propose an improved TLBO algorithm to solve industrial engineering optimization problems. Given the characteristics of TLBO, reinforcement learning (RL) in machine learning is introduced to the learner phase, and enables the algorithm to choose a more suitable learning mode, which can train the search agents to perform more beneficial actions. In addition, a random opposition-based learning (ROBL) strategy is added after the whole learner phase to facilitate the convergence acceleration and avoid local optima. The proposed improved TLBO with RH and ROBL strategies is called RLTLBO. The standard and CEC2017 benchmark functions and eight engineering design problems are used to test the exploration and exploitation capabilities of the proposed method. The RLTLBO algorithm is compared with some existing algorithms, including the basic TLBO and the Salp Swarm Algorithm (SSA), which are considered the classical algorithms, the Aquila Optimizer (AO), Harris Hawks Optimization (HHO) [30], and Horse herd Optimization Algorithm (HOA) [31], which are the recent new methods, and the memory-based Grey Wolf Optimizer (mGWO) [32], modified Ant Lion Optimizer (MALO) [33] and dynamic Sine Cosine Algorithm (DSCA) [34], which are the latest improved algorithms. The experimental results show that the proposed RLTLBO method is superior to the state-of-the-art algorithms in exploration and exploitation capabilities. Moreover, eight industrial engineering design problems are applied to evaluate the effectiveness of the algorithm when solving real-world optimization problems.

The rest of this paper is organized as follows: Section 2 provides a brief overview of the basic TLBO, RL, and ROBL strategies.  Section 3 describes the proposed RLTLBO algorithm in detail. Simulations, experiments and an analysis of the results are presented in Section 4.  Section 5 describes industrial engineering design problems. Finally, Section 6 concludes the paper.

## 2. Related Work

### 2.1. Teaching-Learning-Based Optimization

The TLBO algorithm mimics the influence of a teacher on the output of learners, which can be reflected by learners' grades. As a highly learned person, the teacher gives their knowledge to the learners. The outcome of the learners is affected by the quality of the teacher. It is obvious that learners trained by a good teacher can achieve better results in terms of their grades. The optimization process of TLBO is divided into two phases: the teacher phase and the learner phase.

#### 2.1.1. Teacher Phase

The teacher phase simulates the teaching process of a teacher. The best one in the class is selected as the teacher, and then the teacher tries their best to improve the overall level of the class. The teaching process can be formulated as follows:

(1) $$\begin{array}{c} X_{\text{new}}=X_{\text{old}}+\text{rand}\left(X_{\text{teacher}}-T_{F}\cdot \text{Mean}\right), \end{array}$$

where Xnew and Xold represent the positions of the individual after and before learning, that is, the candidate solutions after and before updating. Xteacher is the position of the teacher, which is the best individual of the population. Mean indicates the average level of search agents in the population. TF is a teaching factor that determines the change of the mean value, and rand is a random number between 0 and 1. The value of TF can be either 1 or 2, which is a heuristic step and randomly decided with equal probability as TF = round (1 + rand (0, 1){2–1}).

#### 2.1.2. Learner Phase

In addition to learning new knowledge from the teacher, learners can also increase knowledge through interaction. In the mutual learning process, a learner can randomly learn knowledge from another learner with a better grade randomly. The expression of the learner phase can be written as follows:

(2) $$\begin{array}{c} X_{\text{new}}=\left\{\begin{array}{c} X_{\text{old}}+\text{rand}\left(X_{r1}-X_{r2}\right)f\left(X_{r1}\right)<f\left(X_{r2}\right)\\ X_{\text{old}}+\text{rand}\left(X_{r2}-X_{r1}\right)\text{otherwise} \end{array}\right., \end{array}$$

where Xr1 and Xr2 indicate the positions of two learners randomly selected from the population. *f* (·) is the fitness value. The comparison between two learners determines the learning direction. The individual with a poor grade learns from the individual with a better grade. The new individual with improvements after learning will be accepted, otherwise rejected.

**Figure 1:**
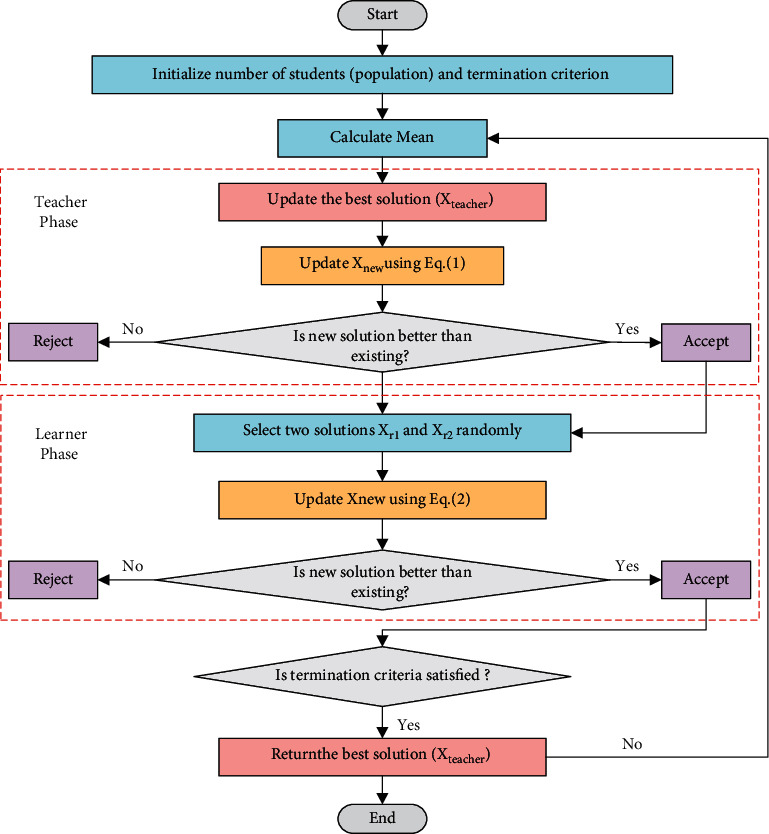
The flowchart of TLBO.

The flow chart of the TLBO algorithm is shown in Figure 1.

### 2.2. Reinforcement Learning (RL)

**Figure 2:**
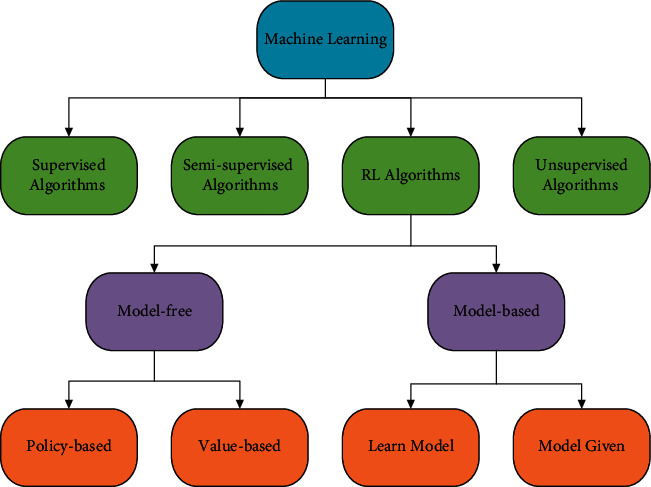
Classification of the reinforcement learning algorithms.

Machine learning algorithms are also widely used to solve various optimization problems [35]. Machine learning methods generally consist of four categories, as shown in Figure 2: supervised learning, unsupervised learning, semisupervised learning, and reinforcement learning (RL). In RL algorithms, the agent is trained to learn optimal actions in a complex environment. The agent is trained in different ways and uses its training experience in the subsequent actions. RL methods generally consist of model-free and model-based approaches. The model-free approaches can be divided into two subgroups: value-based and policy-based methods. The value-based algorithms are convenient for coordinating with meta-heuristic algorithms because they are model-free and policy-free, providing higher flexibility [36]. In the value-based RL approaches, the reinforcement agent learns from its actions and experience in the environment, such through reward and penalty. The agent measures the success of the action in completing the task goal through the reward penalty and then makes a decision based on its achievement.

The Q-Learning method is one of the representative algorithms among the value-based RL methods. In the Q-Learning method, the agent takes random actions and then obtains a reward or penalty. An experience is gradually constructed based on the agent's actions. Throughout process of building experience, a table called Q-Table is defined [37]. The agent considers all possible actions and tries to update its state according to the Q-Table values to select the best action that maximizes the current state's maximal rewards. Therefore, the agent in action determines whether to explore or exploit the environment.

Compared to RL methods, meta-heuristic algorithms often require deep expert knowledge to establish the balance between different phases. RL methods can help discover optimal designs of parameters and more balanced strategies allowing the algorithm to switch between the exploration and exploitation phases. Metaheuristic methods usually operate with specific policies in certain situations, and thus, the dynamism is lower than that of RL algorithms, especially value-based methods. The agent in the value-based methods is online and operates beneficial actions through a reward-penalty mechanism without following any policy. Many types of research have been presented in the literature regarding the combination of meta-heuristics and RL [38–44].

### 2.3. Random Opposition-Based Learning (ROBL)

**Figure 3:**
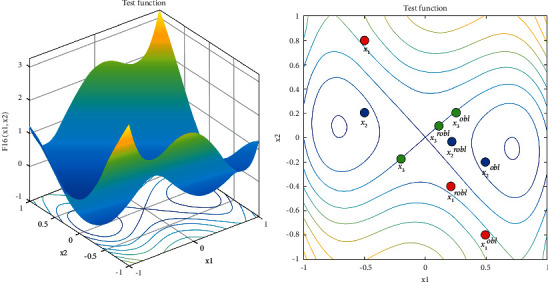
Example of ROBL solutions. Three sets of solutions (original solution, corresponding opposite solution (xobl), and random opposite solution (xrobl)) are labeled in a two-dimensional search space. The random opposite solutions are not only in the symmetric positions, but also with a wider range of distributions.

Random opposition-based learning (ROBL) is a variant of opposition-based learning (OBL) [45] proposed by Long et al. in 2019 [46]. OBL is a powerful optimization tool that simultaneously considers the fitness of an estimate and its corresponding opposite estimate to achieve a better candidate solution. In contrast from the basic OBL, ROBL utilizes a random term to improve the OBL strategy, which is defined as follows:

(3) $$\begin{array}{c} {\hat{x}}_{j}=l_{j}+u_{j}-\text{rand}\times x_{j},\;j=1,\;2,\;\ldots ,\;n, \end{array}$$

where ${\hat{x}}_{j}$ and *x*_*j*_ indicate the opposite and original solutions, *u*_*j*_ and *l*_*j*_ are the upper and lower bound of the problem in jth dimension. The opposite solution is randomly selected in the opposite half of the search space. This solution is not only opposite, but also random, with a wider range of distributions. An example of ROBL solutions is shown in Figure 3. The opposite solution with a random term described by equation (3) is more stochastic than the basic OBL and can effectively help the algorithm jump out of the local optima.

## 3. The Proposed RLTLBO Algorithm

### 3.1. New Learning Mode

The basic TLBO algorithm performs the learner phase after the teacher phase in each iteration. The search agent learns from other individuals in the learner phase. However, in the actual learning process, students learning from each other varies from person to person. Different students might choose different learning modes, such as formal communications, group discussions, presentations, etc. Moreover, the students might adjust the learning mode according to their learning situation during the learning process. Therefore, in this paper, we introduce another learning mode to diversify the learning methods of the students, which can be described in the equations as follows:

(4) $$\begin{array}{c} X_{\text{new}}=\left\{\begin{array}{c} X_{\text{old}}+\text{rand}\left[\left(1-\frac{t}{T}\right)X_{r3}+\left(\frac{t}{T}\right)X_{\text{teacher}}-X_{\text{old}}\right]f\left(X_{r3}\right)<f\left(X_{\text{old}}\right)\\ X_{\text{old}}+\text{rand}\left[\left(1-\frac{t}{T}\right)X_{\text{old}}+\left(\frac{t}{T}\right)X_{\text{teacher}}-X_{r3}\right]\text{otherwise} \end{array}\right., \end{array}$$

where Xr3 is the position of a learner randomly selected from the population. *t* and *T* are the current and maximum number of iterations.

In this learning mode, the effect of the teacher is introduced. Sometimes the mutual learning between students is not always beneficial, and the partial intervention of the teacher is more helpful to students' improvement. Students will not only learn from each other but also ask the teacher for help. At the beginning of the iterations, the weight of mutual learning among students is larger, and the algorithm pays more attention to random learning, which can maintain population diversity and increase global searchability. In the later iteration stage, students consult more from the teacher and approach the teacher, enhancing the algorithms local searchability.

### 3.2. Learner Phase with RL Strategy

**Figure 4:**
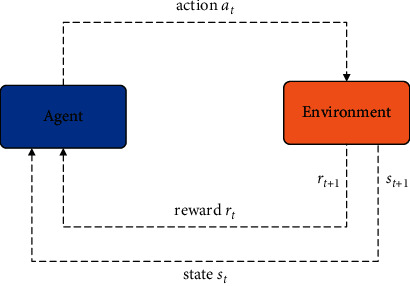
Reinforcement learning agent and environment framework. at represents the current action st and st + 1 indicate the current and the next state, rt and rt + 1 indicate the current and the next reward, respectively.

To enable students to adjust their learning mode more effectively, Q-Learning in RL is introduced to complete the switching between both learning modes. The student uses Q-Table values as a guide to decide between different learning modes. The Q-table is updated using a reward-penalty mechanism. The student selects the best state by calculating the benefit degree of each possible state and taking the leaning mode with the highest Q-values for the next step. The student obtains a reward or a penalty according to its actions after each step. The general pattern of the RL agent and environmental framework is shown in Figure 4.

In the Q-Learning method, a reward table is used to reward or penalize the agent for its action or state compositions, which users can provide. The reward table in this work contains the positive (+1) or negative (−1) rewards for each state and action couple. The Q-Table can be considered the agents experience, which should be assigned a zero value for all units in the beginning. Consequently, the student updates Q-Table using the Bellman equation (5) and prepares the Q-Table for the next iteration [44].

(5) $$\begin{array}{c} Q_{\left(t+1\right)}\left(s_{t},a_{t}\right)\leftarrow Q_{t}\left(s_{t},a_{t}\right)+\lambda \left[r_{t+1}+\gamma \text{Max}Q_{t_{\left(s_{t+1},a\right)}}-Q_{t}\left(s_{t},a_{t}\right)\right], \end{array}$$

where st and st + 1 indicate the current and the next state respectively, Qt and Qt + 1 are the current Q-value and pre-estimated Q-value for the next state st + 1, and at represents the current action. *λ* and *γ* are the learning rate value and discount factor, respectively, which are numbers between 0 and 1. The learning rate determines how fast the algorithm should learn and controls the convergence of the learning process. The discount factor defines how much the algorithm learns from the mistake and controls the importance of future rewards. rt + 1 indicates the immediate reward or penalty an agent gets for taking current action.

**Figure 5:**
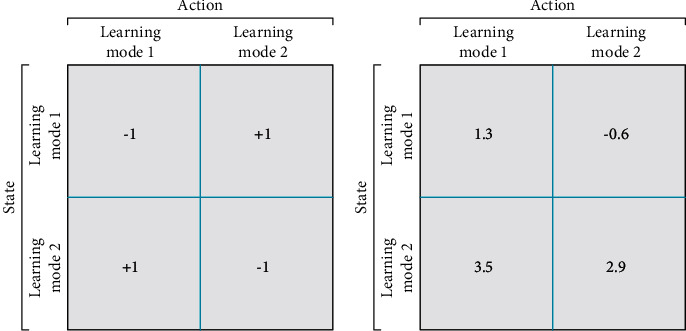
The reward table and Q-Table example of RLTLBO. (a) Reward Table sample (b) Q-Table sample.

In each iteration, the agent uses equation (5) to calculate and weight each possible state and action for the next step, before choosing the best action (learning mode 1 or learning mode (2) with the highest likelihood to get closer to the best optimal solution. Examples of the reward table and Q-Table are displayed in Figure 5. This RL strategy helps establish a switching mechanism between different learning modes in the learner phase and find the most suitable decision scheme. Four optional actions can occur as listed below:

1. When the student is learning in learning mode 1, they still decides to stay in learning mode 1
2. When the student is learning in learning mode 2, they still decides to stay in learning mode 2
3. When the student learns in learning mode 1, they decides to transition to learning mode 2
4. When the student learns in learning mode 1, they decides to transition to learning mode 2

The most important value of the RL strategy is to help the algorithm switch between different learning modes as and when needed during the learner phase. For the above reason, the algorithm can find better solutions faster and more effectively in the search space, considerably increasing the search efficiency. Therefore, the convergence speed of the algorithm can be improved effectively.

### 3.3. The Detail of RLTLBO

**Figure 6:**
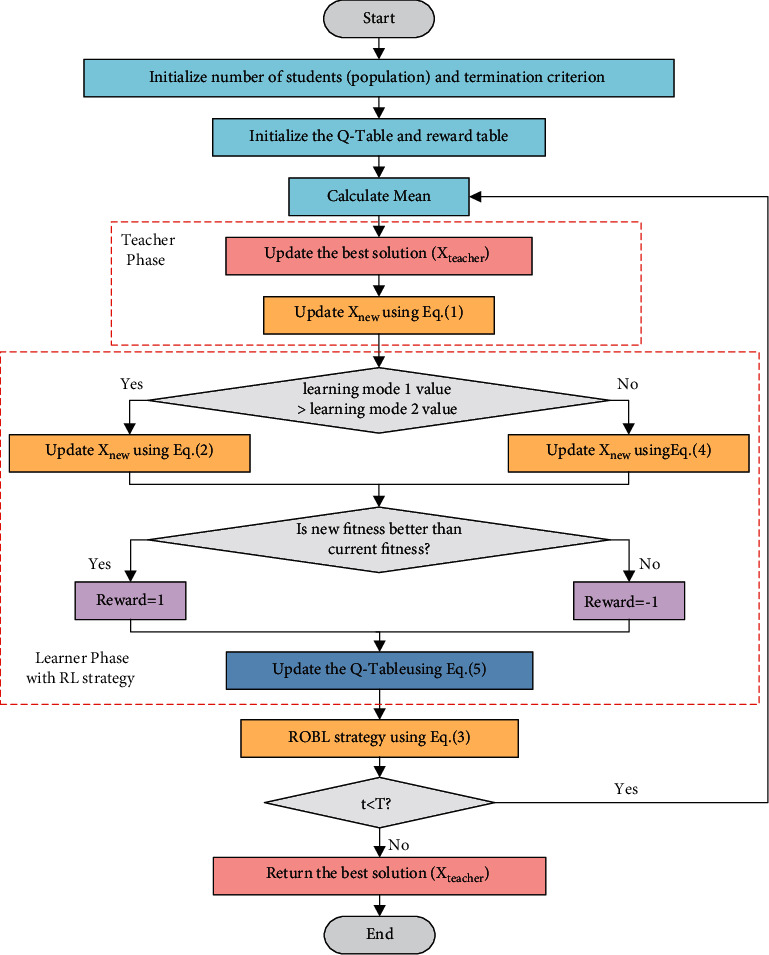
The flowchart of RLTLBO.

In the improved TLBO algorithm, the teacher phase of basic TLBO is carried out first. Then, the learner phase with RL strategy is implemented to achieve effective and efficient investigation in the search space. Finally, ROBL is added to enhance the ability of local optima avoidability. The random opposite solution increases the probability of the algorithm finding a better solution. This variant of TLBO, which incorporates RL, is named RLTLBO. The pseudocode and the flowchart of the proposed RLTLBO algorithm are shown in Algorithm 1 and Figure 6, respectively.

### 3.4. Computational Complexity Analysis

RLTLBO mainly consists of three components: initialization, fitness evaluation, and position updating. In the initialization phase, the computational complexity of positions generated is O(N). Then, the computational complexity of fitness evaluation for the solution is O(2 × N) during the iteration process. Finally, we utilize ROBL to keep the algorithm from falling into local optima. Thus, the computational complexities of position updating of RLTLBO is O(2 × *N* × *D*), where *D* is the dimension size of the problem. Therefore, the total computational complexity of the proposed RLTLBO algorithm is O(3 × *N* + 2 × *N* × *D*).

## 4. Numerical Experiments and Results

In this section, two different kinds of benchmark functions are performed to evaluate the performance of the proposed RLTLBO algorithm. Standard benchmark functions are first tested to assess the algorithm in solving twenty-three simple numerical problems. Then, the CEC2017 benchmark functions are utilized to evaluate the algorithm in solving complex numerical problems. The RLTLBO is compared with three types of existing algorithms, including the classic methods, TLBO and SSA, the recently proposed algorithms, HOA [31], AO, and HHO [30], and the improved algorithms, mGWO [32], MALO [33] and DSCA [34]. For the consistency of all tests, we set the population size to *N* = 30, the dimension size to *D* = 30, and the maximum number of iterations to *T* = 500. All algorithms are run 30 times independently, and the average values and standard deviations are presented as the final experimental results. All experiments are implemented in MATLAB R2020b on a PC with Intel (*R*) Core (TM) i5-9500 CPU @ 3.00 GHz and RAM 16 GB memory on OS windows 10.

### 4.1. Standard Benchmark Function Experiments

Standard benchmark functions [47] can be divided into three types: unimodal, multimodal and fixed-dimension multimodal functions. Unimodal functions only have one global optimum and no local optima, which can be used to evaluate an algorithm's convergence rate and exploitation capability. Multimodal and fixed-dimension multimodal functions have a global optimum and multiple local optima. This characteristic makes these functions effective for testing the exploration and local optima avoidance abilities of an algorithm. The benchmark function details are listed in Tables 1–3.

#### 4.1.1. Qualitative Results

The data results of the 23 standard benchmark functions are shown in Table 4, and the optimal results are bolded. For the unimodal functions F1–F7, the RLTLBO algorithm achieves the best results among all comparative algorithms on most functions in average values and standard deviations, and only obtains worse results on F5–F6. The RLTLBO obtains the theoretical optimum of F1 and F3. It can be concluded from the comparison results that RLTLBO has strong competitiveness in the unimodal functions, which indicates that the excellent exploitation capability comes from the RL mechanism.

For the multimodal and fixed-dimension multimodal functions F8–F23, it can be seen from Table 4 that RLTLBO achieves the smallest average values and standard deviations on 12 of all 16 test functions compared to other methods, which indicates a very high accuracy and stability. Several poor results appear on F8 and F12–F14, but they are not the worst results. The satisfying results on the multimodal and fixed-dimension multimodal functions prove that the exploration and local optima avoidance capabilities of the RLTLBO are excellent, which might be derived from the ROBL strategy.

**Figure 7:**
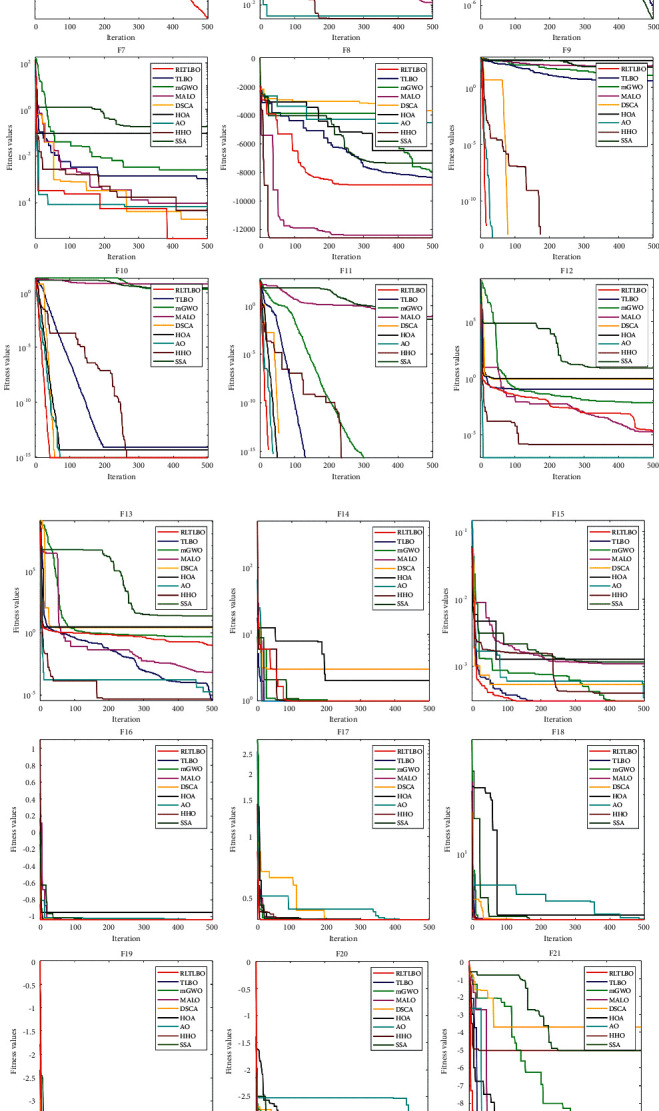
Convergence curves of 23 standard benchmark functions.

Figure 7 provides the convergence curves of RLTLBO and the comparative algorithms for 23 standard benchmark functions. The convergence rate reflected by convergence curves can show us the improvement of exploration and exploitation more intuitively. For F1–F4, F7, F9–F11, and F15–F21, the RLTLBO presents a faster convergence speed than other meta-heuristic algorithms, and the convergence accuracy is also the best. The RLTLBO is ranked in the second position in terms of convergence speed for F22 and F23. For benchmark functions F5–F6, F8, and F12–F14, the RLTLBO does not perform very well, the same as the results in Table 4.

#### 4.1.2. The Wilcoxon Test

The Wilcoxon rank-sum test [48] results are listed in Table 5, which can assess the statistical performance differences between the RLTLBO algorithm and the comparative algorithms. A *p*-value less than 0.05 indicates a substantial difference between the two compared methods. It is obvious that the overwhelming majority *p*-values in Table 5 are less than 0.05, indicating that there are statistically and substantial differences between RLTLBO and the other methods. Combining the results in Table 4, it can be concluded that the RLTLBO algorithm outperforms the others. The competitive results of RLTLBO indicate that this algorithm has high capabilities of exploration and exploitation. In summary, the RLTLBO algorithm provides better results than other comparative algorithms.

### 4.2. CEC2017 Benchmark Function Experiments

Standard benchmark function experiments prove the superior performance on simple optimization problems of the proposed RLTLBO algorithm. CEC2017 [49], one of the most challenging test suites, can help check the performance of complex optimization problems. Some hybrid and composition functions are selected to further test the performance of RLTLBO. These types of functions are precisely what the standard test functions do not have. The functional details and the comparison results are presented in Tables 6 and 7. As mentioned above, each method runs 30 times with 30 search agents and 500 iterations. From Table 7, the proposed RLTLBO achieves both the best average and standard deviation values on five of the eight all functions. For the remaining three functions, RLTLBO obtains one of the best average and standard deviation values. The RLTLBO completely exceeds the TLBO, MALO, HOA, AO, HHO, and SSA methods completely. The statistical results are also listed in Table 8. There are only seven *p*-values greater than 0.05 in all test functions, which means considerable differences between the RLTLBO and the compared methods. These results suggest that RLTLBO can achieve great results on complex problems as well.

## 5. Experiments on Industrial Engineering Design Problems

In this section, eight well-known constrained industrial engineering design problems, including the welded beam design problem, pressure vessel design problem, tension and compression spring design problem, speed reducer design problem, three-bar truss design problem, car crashworthiness design problem, tubular column design problem, and frequency-modulated sound wave design problem, are solved to further verify the performance of the proposed RLTLBO algorithm. The results of RLTLBO are compared to various optimization methods proposed in previous studies.

### 5.1. Welded Beam Design Problem

**Figure 8:**
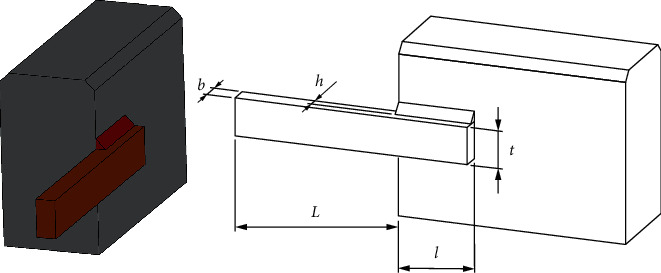
Welded beam design problem.

The purpose of this problem is to minimize the cost of the welded beam (Figure 8). Four variables need to be optimized: the thickness of weld (h), the thickness of the bar (b), length of the bar (l), and height of the bar (*t*). The mathematical formulation is listed as follows:

Consider $\overset{⟶}{z}=\left[z_{1},z_{2},z_{3},z_{4}\right]=\left[h,l,t,b\right].$
Minimize $f\left(\overset{⟶}{z}\right)=1.10471z_{1}^{2}z_{2}+0.04811z_{3}z_{4}\left(14.0+z_{2}\right),$  subject to
  (6) $$\begin{array}{c} \left\{\begin{array}{c} g_{1}\left(\overset{⟶}{z}\right)=\tau \left(\overset{⟶}{z}\right)-\tau_{\max}\leq 0,\\ g_{2}\left(\overset{⟶}{z}\right)=\sigma \left(\overset{⟶}{z}\right)-\sigma_{\max}\leq 0,\\ g_{3}\left(\overset{⟶}{z}\right)=\delta \left(\overset{⟶}{z}\right)-\delta_{\max}\leq 0,\\ g_{4}\left(\overset{⟶}{z}\right)=z_{1}-z_{4}\leq 0,\\ g_{5}\left(\overset{⟶}{z}\right)=P-P_{c}\left(\overset{⟶}{z}\right)\leq 0,\\ g_{6}\left(\overset{⟶}{z}\right)=0.125-z_{1}\leq 0,\\ g_{7}\left(\overset{⟶}{z}\right)=1.10471z_{1}^{2}+0.04811z_{3}z_{4}\left(14.0+z_{2}\right)-5.0\leq 0. \end{array}\right. \end{array}$$
Variable range

(7) $$\begin{array}{c} \left\{\begin{array}{c} 0.1\leq z_{1}\leq 2,\\ 0.1\leq z_{2}\leq 10,\\ 0.1\leq z_{3}\leq 10,\\ 0.1\leq z_{4}\leq 2, \end{array}\right. \end{array}$$

where

(8) $$\begin{array}{c} \left\{\begin{array}{c} \tau \left(\overset{⟶}{z}\right)=\sqrt{{\tau^{\prime }}^{2}+2\tau^{\prime }\tau^{\prime \prime }\frac{z_{2}}{2R}+{\tau^{\prime \prime }}^{2}},\\ \tau^{\prime }=\frac{P}{\sqrt{2}z_{1}z_{2}},\tau^{\prime \prime }=\frac{\text{MR}}{J},M=P\left(L+\frac{z_{2}}{2}\right),\\ R=\sqrt{\frac{z_{2}^{2}}{4}+{\left(\frac{z_{1}+z_{3}}{2}\right)}^{2}},\\ J=2\left\{\sqrt{2}z_{1}z_{2}\left[\frac{z_{2}^{2}}{12}+{\left(\frac{z_{1}+z_{3}}{2}\right)}^{2}\right]\right\},\\ \sigma \left(\overset{⟶}{z}\right)=\frac{6PL}{z_{4}z_{3}^{2}},\delta \left(\overset{⟶}{z}\right)=\frac{4PL^{3}}{Ez_{3}^{3}z_{4}},\\ P_{c}\left(\overset{⟶}{z}\right)=\frac{4.013E\sqrt{z_{3}^{2}z_{4}^{6}/36}}{L^{2}}\left(1-\frac{z_{3}}{2L}\sqrt{\frac{E}{4G}}\right),\\ P=6000lb,L=14\text{in},\delta_{max}=0.25\text{in,}\\ E=30\times 10^{6}psi,G=12\times 10^{6}psi,\\ \tau_{max}=13600psi,\sigma_{max}=30000psi. \end{array}\right. \end{array}$$

The RLTLBO is compared to SMA [50], WOA, MPA [51], MVO [52], GA, and HS [53] methods. The comparison results presented in Table 9 show the superior of the RLTLBO algorithm with a smaller cost than other algorithms.

### 5.2. Pressure Vessel Design Problem

**Figure 9:**
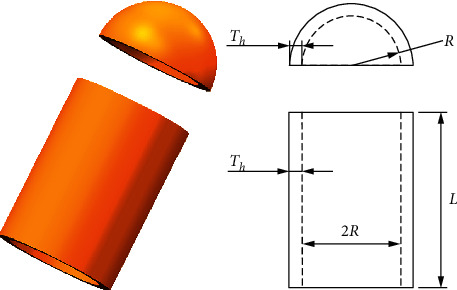
Pressure vessel design problem.

The objective of this problem is to minimize the fabrication cost of the cylindrical pressure vessel to meet the pressure requirements. As shown in Figure 9, four structural parameters in this problem need to be minimized, including the thickness of the shell (Ts), the thickness of the head (Th), inner radius (R), and the length of the cylindrical section without the head (*L*). The formulation of four optimization constraints can be described as follows:

Consider $\overset{⟶}{x}=\left[x_{1}\;x_{2}\;x_{3}\;x_{4}\right]=\left[T_{s}\;T_{h}\;R\;L\right].$
Minimize $f\left(\overset{⟶}{x}\right)=0.6224x_{1}x_{3}x_{4}+1.7781x_{2}x_{3}^{2}+3.1661x_{1}^{2}x_{4}+19.84x_{1}^{2}x_{3},$ subject to
  (9) $$\begin{array}{c} \left\{\begin{array}{c} g_{1}\left(\overset{⟶}{x}\right)=-x_{1}+0.0193x_{3}\leq 0,\\ g_{2}\left(\overset{⟶}{x}\right)=-x_{3}+0.00954x_{3}\leq 0,\\ g_{3}\left(\overset{⟶}{x}\right)=-\pi x_{3}^{2}x_{4}-\frac{4}{3}\pi x_{3}^{3}+1296000\leq 0,\\ g_{4}\left(\overset{⟶}{x}\right)=x_{4}-240\leq 0. \end{array}\right. \end{array}$$
Variable range
  (10) $$\begin{array}{c} \left\{\begin{array}{c} 0\leq x_{1}\leq 99,\\ 0\leq x_{2}\leq 99,\\ 10\leq x_{3}\leq 200,\\ 10\leq x_{4}\leq 200. \end{array}\right. \end{array}$$

From the results in Table 10, it is obvious that RLTLBO can obtain superior optimal values compared to AO, SMA, WOA, GWO, MVO, GA, and ES [54].

### 5.3. Tension/Compression Spring Design Problem

**Figure 10:**
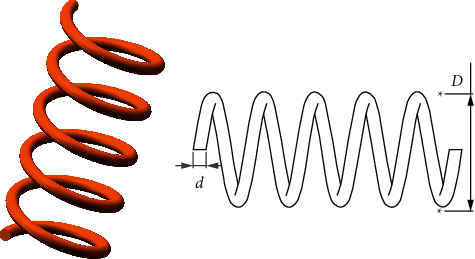
Tension/compression spring design problem.

This problem aims to minimize the weight of the tension/compression spring (Figure 10). Three variables need to be optimized, including the wire diameter (d), the number of active coils (N), and mean coil diameter (D). This problem can be described as follows:

Consider $\overset{⟶}{x}=\left[x_{1}\;x_{2}\;x_{3}\right]=\left[d\;D\;N\right].$
Minimize $f\left(\overset{⟶}{x}\right)=\left(x_{3}+2\right)x_{2}x_{1}^{2},$  subject to
  (11) $$\begin{array}{c} \left\{\begin{array}{c} g_{1}\left(\overset{⟶}{x}\right)=1-\frac{x_{2}^{3}x_{3}}{71785x_{1}^{4}}\leq 0,\\ g_{2}\left(\overset{⟶}{x}\right)=\frac{4x_{2}^{2}-x_{1}x_{2}}{12566\left(x_{2}x_{1}^{3}-x_{1}^{4}\right)}+\frac{1}{5108x_{1}^{2}}\leq 0,\\ g_{3}\left(\overset{⟶}{x}\right)=1-\frac{140.45x_{1}}{x_{2}^{2}x_{3}}\leq 0,\\ g_{4}\left(\overset{⟶}{x}\right)=\frac{x_{1}+x_{2}}{1.5}-1\leq 0. \end{array}\right. \end{array}$$
Variable range
  (12) $$\begin{array}{c} \left\{\begin{array}{c} 0.05\leq x_{1}\leq 2.00,\\ 0.25\leq x_{2}\leq 1.30,\\ 2.00\leq x_{3}\leq 15.00. \end{array}\right. \end{array}$$

The RLTLBO is compared to AO, SSA, WOA, GWO, PSO, GA, and HS algorithms. Results are listed in Table 11 and show that the RLTLBO can obtain the best weight compared to all other algorithms.

### 5.4. Speed Reducer Design Problem

**Figure 11:**
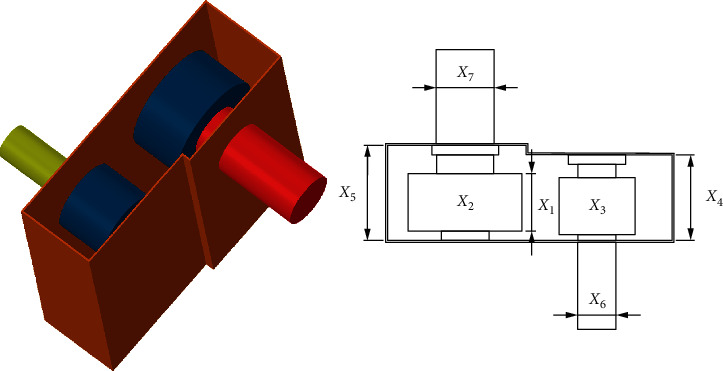
Speed reducer design problem.

In this case, the purpose is to minimize the weight of the speed reducer (Figure 11). Seven variables are considered, including face width (x1), a module of teeth (x2), a discrete design variable on behalf of the teeth in the pinion (x3), length of the first shaft between bearings (x4), length of the second shaft between bearings (x5), diameters of the first shaft (x6), and diameters of the second shaft (x7). The mathematical formulation is listed as follows:

Minimize

(13) $$\begin{array}{c} f\left(\overset{⟶}{x}\right)=\left.0.7854x_{1}x_{2}^{2}+3.3333x_{3}^{2}+14.9334x_{3}-43.0934\right)-1.508x_{1}\left(x_{6}^{2}+x_{7}^{2}\right)+7.4777\left(x_{6}^{3}+x_{7}^{3}\right), \end{array}$$

subject to

(14) $$\begin{array}{c} \left\{\begin{array}{c} g_{1}\left(\overset{⟶}{x}\right)=\frac{27}{x_{1}x_{2}^{2}x_{3}}-1\leq 0,\\ g_{2}\left(\overset{⟶}{x}\right)=\frac{397.5}{x_{1}x_{2}^{2}x_{3}^{2}}-1\leq 0,\\ g_{3}\left(\overset{⟶}{x}\right)=\frac{1.93x_{4}^{3}}{x_{2}x_{3}x_{6}^{4}}-1\leq 0,\\ g_{4}\left(\overset{⟶}{x}\right)=\frac{1.93x_{5}^{3}}{x_{2}x_{3}x_{7}^{4}}-1\leq 0,\\ g_{5}\left(\overset{⟶}{x}\right)=\frac{\sqrt{{\left(745x_{4}/x_{2}x_{3}\right)}^{2}+16.9\times 10^{6}}}{110.0x_{6}^{3}}-1\leq 0,\\ g_{6}\left(\overset{⟶}{x}\right)=\frac{\sqrt{{\left(745x_{4}/x_{2}x_{3}\right)}^{2}+157.5\times 10^{6}}}{85.0x_{6}^{3}}-1\leq 0,\\ g_{7}\left(\overset{⟶}{x}\right)=\frac{x_{2}x_{3}}{40}-1\leq 0,\\ g_{8}\left(\overset{⟶}{x}\right)=\frac{5x_{2}}{x_{1}}-1\leq 0,\\ g_{9}\left(\overset{⟶}{x}\right)=\frac{x_{1}}{12x_{2}}-1\leq 0,\\ g_{10}\left(\overset{⟶}{x}\right)=\frac{1.5x_{6}+1.9}{x_{4}}-1\leq 0,\\ g_{11}\left(\overset{⟶}{x}\right)=\frac{1.1x_{7}+1.9}{x_{5}}-1\leq 0. \end{array}\right. \end{array}$$

Variable range

(15) $$\begin{array}{c} \left\{\begin{array}{c} 2.6\leq x_{1}\leq 3.6,\\ 0.7\leq x_{2}\leq 0.8,\\ 17\leq x_{3}\leq 28,\\ 7.3\leq x_{4}\leq 8.3,\\ 7.8\leq x_{5}\leq 8.3,\\ 2.9\leq x_{6}\leq 3.9,\\ 5.0\leq x_{7}\leq 5.5. \end{array}\right. \end{array}$$

Compared to AO, PSO, AOA, GA, SCA [55], HS, and FA [56], RLTLBO achieves better results in the speed reducer problem, as shown in Table 12.

### 5.5. Three-Bar Truss Design Problem

**Figure 12:**
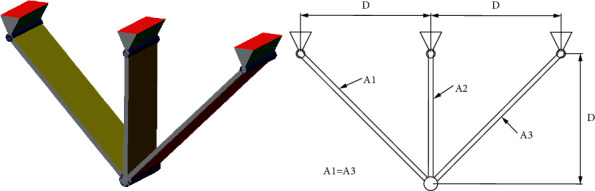
Thre*E − *bar truss design problem.

The three-bar truss design problem aims to minimize the weight of a truss with three bars by controlling the length of three bars (A1, A2, and A3) (Figure 12). Three main constraints need to be satisfied, including deflection, stress, and buckling. The mathematical form of this problem is given:

Consider $\overset{⟶}{x}=\left[x_{1}\;x_{2}\right]=\left[A_{1}\;A_{2}\right].$
Minimize $f\left(\overset{⟶}{x}\right)=\left(2\sqrt{2}x_{1}+x_{2}\right)*l$  subject to
  (16) $$\begin{array}{c} \left\{\begin{array}{c} g_{1}\left(\overset{⟶}{x}\right)=\frac{\sqrt{2}x_{1}+x_{2}}{\sqrt{2}x_{1}^{2}+2x_{1}x_{2}}P-\sigma \leq 0,\\ g_{2}\left(\overset{⟶}{x}\right)=\frac{x_{2}}{\sqrt{2}x_{1}^{2}+2x_{1}x_{2}}P-\sigma \leq 0,\\ g_{3}\left(\overset{⟶}{x}\right)=\frac{1}{\sqrt{2}x_{2}+x_{1}}P-\sigma \leq 0. \end{array}\right. \end{array}$$
Consider 0 ≤ *x*_1_, *x*_2_ ≤ 1, where *l*=100cm, *P*=2*KN*/*cm*^2^, *σ*=2*KN*/*cm*^2^.

The result of RLTLBO is listed in Table 13, compared to AO, SSA, AOA, MVO, and GOA [57]. It can be observed that RLTLBO outperforms other algorithms in the literature.

### 5.6. Car Crashworthiness Design Problem

The car crashworthiness design problem aims to minimize the weight by optimizing eleven influence variables [58], including the thickness of B-Pillar inner (x1), B-pillar reinforcement (x2), floor side inner (x3), cross members (x4), door beam (x5), door beltline reinforcement (x6) and roof rail (x7), materials of B-Pillar inner (x8) and floor side inner (x9), barrier height (x10), and barrier hitting position (x11). This problem can be formulated as follows.

Minimize

(17) $$\begin{array}{c} f\left(\overset{⟶}{x}\right)=\text{weight}, \end{array}$$

subject to

(18) $$\begin{array}{c} \left\{\begin{array}{c} g_{1}\left(\overset{⟶}{x}\right)=F_{a}\left(\text{load in abdomen}\right)\leq 1\;kN,\\ g_{2}\left(\overset{⟶}{x}\right)=V\times Cu\left(\text{dummy upper chest}\right)\leq 0.32m/s,\\ g_{3}\left(\overset{⟶}{x}\right)=V\times Cm\left(\text{dummy middle chest}\right)\leq 0.32m/s,\\ g_{4}\left(\overset{⟶}{x}\right)=V\times Cl\left(\text{dummy lowere chest}\right)\leq 0.32m/s,\\ g_{5}\left(\overset{⟶}{x}\right)=\Delta_{ur}\left(\text{upper rib deflection}\right)\leq 32\text{mm},\\ g_{6}\left(\overset{⟶}{x}\right)=\Delta_{mr}\left(\text{middle rib deflection}\right)\leq 32\text{mm},\\ g_{7}\left(\overset{⟶}{x}\right)=\Delta_{lr}\left(\text{lower rib deflection}\right)\leq 32\text{mm},\\ g_{8}\left(\overset{⟶}{x}\right)=F{\left(\text{public force}\right)}_{p}\leq 4kN,\\ g_{9}\left(\overset{⟶}{x}\right)=V_{\text{MBP}}\left(\text{v}elocity\;of\;V-\text{pillar }at\;middle\;point\right)\leq 9.9\text{mm}/ms,\\ g_{10}\left(\overset{⟶}{x}\right)=V_{\text{FD}}\left(\text{v}elocity\;of\;front\;doorat\;V-\text{pillar}\right)\leq 15.7\text{mm}/ms. \end{array}\right.. \end{array}$$

Variable range

(19) $$\begin{array}{c} \left\{\begin{array}{c} 0.5\leq x_{1}-x_{7}\leq 1.5,\\ x_{8},x_{9}\in \left(0.192,0.345\right),\\ -30\leq x_{10},x_{11}\leq 30. \end{array}\right. \end{array}$$

The RLTLBO and DE, GA, FA, CS [59], GOA, and EOBL-GOA [58] are applied to solve the car crashworthiness problem. As shown in Table 14, compared to other methods, the proposed RLTLBO achieves the best result than others.

### 5.7. Tubular Column Design Problem

**Figure 13:**
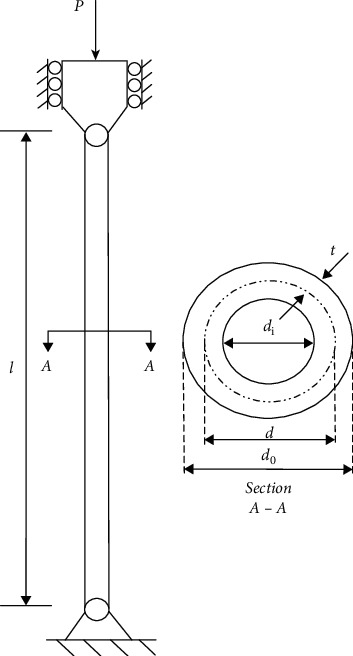
Tubular column design problem [59].

The main intention is to find a minimum cost for a uniform column, making the tubular section be able to carry a compressive load *P* = 2,500 kgf. The column is made of a material with a yield stress (*σ*y) of 500 kgf/cm^2^, a modulus of elasticity (*E*) of 0.85 × 106 kgf/cm^2^, and a density (*ρ*) equal to 0.0025 kgf/cm^3^. The length (*L*) of the column is 250 cm. The cost of the column consists of material and construction costs. This problem is shown in Figure 13, and the optimization model of the problem is listed as follows.

Minimize *f*(*d*, *t*)=9.8d*t*+2  *d* subject to

(20) $$\begin{array}{c} \left\{\begin{array}{c} g_{1}=\frac{P}{\pi \text{d}t\sigma_{y}}-1\leq 0,\\ g_{2}=\frac{8PL^{2}}{\pi^{3}E\text{d}t\left(d^{2}+t^{2}\right)}-1\leq 0,\\ g_{3}=\frac{2.0}{d}-1\leq 0,\\ g_{4}=\frac{d}{14}-1\leq 0,\\ g_{5}=\frac{0.2}{t}-1\leq 0,\\ g_{6}=\frac{t}{0.8}-1\leq 0. \end{array}\right.. \end{array}$$

From the comparison results in Table 15, we can see that RLTLBO can obtain superior optimal cost compared to mGWO, DSCA, HOA, AO, HHO, and CS.

### 5.8. Frequency-Modulated Sound Waves Design Problem

This problem aims to optimize the frequency-modulated (FM) synthesizer parameter in six dimensions [60]. The following equation is given for optimization *X*={*a*_1_, *ω*_1_, *a*_2_*ω*_2_, *a*_3_, *ω*_3_} as a sound wave, where ai (*i* = 1, 2, 3) is the amplitude and *ω*i (*i* = 1, 2, 3) is the angular frequency. This problem has the lowest value $f\left({\overset{⟶}{X}}_{\text{sol}}\right)=0$. The objective function is calculated based on the square errors between the target wave and the estimated wave. This problem is modeled as follows.

Minimize

(21) $$\begin{array}{c} f\left(\overset{⟶}{X}\right)=\sum_{t=0}^{100}{\left(y\left(t\right)-y_{0}\left(t\right)\right)}^{2}, \end{array}$$

where

(22) $$\begin{array}{c} \left\{\begin{array}{c} y\left(t\right)=a_{1}\cdot \;\sin\left(\omega_{1}\cdot t\cdot \theta +a_{2}\cdot \;\sin\left(\omega_{2}\cdot t\cdot \theta +a_{3}\cdot \;\sin\left(\omega_{3}\cdot t\cdot \theta \right)\right)\right),\\ y_{0}\left(t\right)=\left(1.0\right)\cdot \;\sin\left(\left(5.0\right)\cdot t\cdot \theta -\left(1.5\right)\cdot \;\sin\left(\left(4.8\right)\right.\cdot t\cdot \theta +\left(2.0\right)\cdot \;\sin\left(\left(4.9\right)\cdot \left.t\cdot \theta \right)\right)\right),\\ \theta =\frac{2\pi }{100}. \end{array}\right. \end{array}$$

The RLTLBO is compared with GWO, MFO [61], PSO, TSA [62], and FFA [63] algorithms, and the comparison results are listed in Table 16. It is obvious that the proposed method found a much better solution than the comparative algorithms.

In general, the excellent performance in solving industrial engineering design problems suggests that RLTLBO can be widely used in real-world optimization problems.

## 6. Conclusion

This study presents an improved teaching-learning-based optimization algorithm (RLTLBO) by incorporating reinforcement learning (RL) and random opposition-based learning (ROBL) strategies. Because of the defect of the insufficient learning processes, a new learning model is proposed in the learner phase. The two different modes uniting the inherent learning mode are switched through the Q-learning mechanism in RL. This mechanism helps the individuals learn thoroughly, resulting in accelerating the convergence speed of the RLTLBO. To improve the ability of local optima avoidance, the ROBL strategy is appended after the teacher and learner phases. The proposed RLTLBO algorithm is tested using 23 standard and eight CEC2017 benchmark functions to analyze its search performance. Experimental results illustrate competitive results compared to other state-of-the-art meta-heuristic algorithms. To further verify the superiority of RLTLBO, eight industrial engineering design problems are solved. The results are also very competitive with other comparative algorithms.

The code for RLTLBO is provided at https://github.com/WangShuang92/RLTLBO and can be used for more practical problems. However, this algorithm still suffers with premature convergence on several benchmark functions, which can be studied in the future. Moreover, RLTLBO can only solve single objective problems. For future research, binary and multiobjective versions of RLTLBO can be considered. More applications of this algorithm in different fields are valuable works, including text clustering, scheduling problems, appliances management, parameter estimation, feature selection, test classification, image segmentation problems, network applications, sentiment analysis, etc.

## Figures and Tables

**Algorithm 1:**
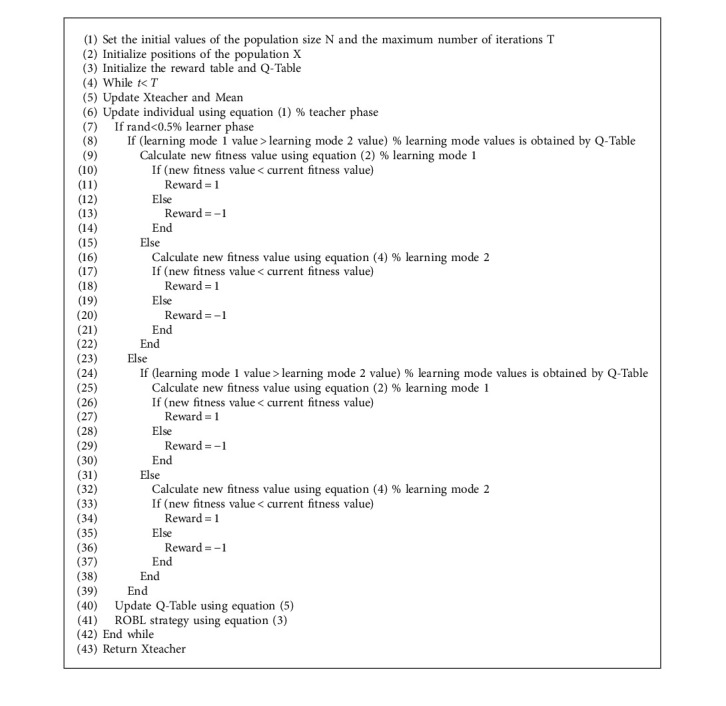
Pseudocode of RLTLBO.

**Table 1:** Unimodal benchmark functions.

Function	Dim	Range	*f*_min_
*F*_1_(*x*)=∑_*i*=1_^*n*^*x*_*i*_^2^	30	[−100, 100]	0
*F*_2_(*x*)=∑_*i*=1_^*n*^|*x*_*i*_|+∏_*i*=1_^*n*^|*x*_*i*_|	30	[−10, 10]	0
*F*_3_(*x*)=∑_*i*=1_^*n*^(∑_*j*−1_^*i*^*x*_*j*_)^2^	30	[−100, 100]	0
*F*_4_(*x*)=max_*i*_{|*x*_*i*_|, 1 ≤ *i* ≤ *n*}	30	[−100, 100]	0
*F*_5_(*x*)=∑_*i*=1_^*n*−1^[100(*x*_*i*+1_ − *x*_*i*_^2^)^2^+(*x*_*i*_ − 1)^2^]	30	[−30, 30]	0
*F*_6_(*x*)=∑_*i*=1_^*n*^(*x*_*i*_+5)^2^	30	[−100, 100]	0
*F*_7_(*x*)=∑_*i*=1_^*n*^*ix*_*i*_^4^+random[0,1)	30	[−1.28, 1.28]	0

**Table 2:** Multimodal benchmark functions.

Function	Dim	Range	*f*_min_
$F_{8}\left(x\right)=\sum_{i=1}^{n}-x_{i}\sin\left(\sqrt{\left|x_{i}\right|}\right)$	30	[−500, 500]	−418.9829 × Dim
*F*_9_(*x*)=∑_*i*=1_^*n*^[*x*_*i*_^2^ − 10 cos(2*πx*_*i*_)+10]	30	[−5.12, 5.12]	0
$F_{10}\left(x\right)=-20\;\exp\left(-0.2\sqrt{1/n\sum_{i=1}^{n}x_{i}^{2}}\right)-\exp\left(1/n\sum_{i=1}^{n}\cos\left(2\pi x_{i}\right)\right)+20+e$	30	[−32, 32]	0
$F_{11}\left(x\right)=1/4000\sum_{i=1}^{n}x_{i}^{2}-\prod_{i=1}^{n}\cos\left(x_{i}/\sqrt{i}\right)+1$	30	[−600, 600]	0
$\begin{array}{c} F_{12}\left(x\right)=\frac{\pi }{n}\left\{10\;\sin\left(\pi y_{1}\right)+{\sum_{i=1}^{n-1}\left(y_{i}-1\right)}^{2}\left[1+10\;\sin^{2}\left(\pi y_{i+1}\right)\right]+{\left(y_{n}-1\right)}^{2}\right\}\\ +\sum_{i=1}^{n}u\left(x_{i},10,100,4\right)\text{,where }y_{i}=1+x_{i}+1/4,\\ u\left(x_{i},a,k,m\right)=\left\{\begin{array}{cc} k{\left(x_{i}-a\right)}^{m} & x_{i}>a\\ 0 & -a<x_{i}<a\\ k{\left(-x_{i}-a\right)}^{m} & x_{i}<-a \end{array}\right. \end{array}$	30	[−50, 50]	0
$\begin{array}{c} F_{13}\left(x\right)=0.1\left(\sin^{2}\left(3\pi x_{1}\right)+\sum_{i=1}^{n}{\left(x_{i}-1\right)}^{2}\left[1+\;\sin^{2}\left(3\pi x_{i}+1\right)\right]+{\left(x_{n}-1\right)}^{2}\left[1+\;\sin^{2}\left(2\pi x_{n}\right)\right]\right)\\ +\sum_{i=1}^{n}u\left(x_{i},5,100,4\right) \end{array}$	30	[−50, 50]	0

**Table 3:** Fixed-dimension multimodal benchmark functions.

Function	Dim	Range	*f*_min_
*F*_14_(*x*)=(1/500+∑_*j*=1_^25^1/*j*+∑_*i*=1_^2^(*x*_*i*_ − *a*_*ij*_)^6^)^−1^	2	[−65, 65]	0.998
*F*_15_(*x*)=∑_*i*=1_^11^[*a*_*i*_ − *x*_1_(*b*_*i*_^2^+*b*_*i*_*x*_2_)/*b*_*i*_^2^+*b*_*i*_*x*_3_+*x*_4_]^2^	4	[−5, 5]	0.00030
*F*_16_(*x*)=4*x*_1_^2^ − 2.1*x*_1_^4^+1/3*x*_1_^6^+*x*_1_*x*_2_ − 4*x*_2_^2^+*x*_2_^4^	2	[−5, 5]	−1.0316
*F*_17_(*x*)=(*x*_2_ − 5.1/4*π*^2^*x*_1_^2^+5/*πx*_1_ − 6)^2^+10(1 − 1/8*π*)cos *x*_1_+10	2	[−5, 5]	0.398
$\begin{array}{c} F_{18}\left(x\right)=\left[1+{\left(x_{1}+x_{2}+1\right)}^{2}\left(19-14x_{1}+3x_{1}^{2}-14x_{2}+6x_{1}x_{2}+3x_{2}^{2}\right)\right]\\ \times \left[30+{\left(2x_{1}-3x_{2}\right)}^{2}\times \left(18-32x_{2}+12x_{1}^{2}+48x_{2}-36x_{1}x_{2}+27x_{2}^{2}\right)\right] \end{array}$	2	[−2, 2]	3
*F*_19_(*x*)=−∑_*i*=1_^4^*c*_*i*_exp(−∑_*j*=1_^3^*a*_*ij*_(*x*_*j*_ − *p*_*ij*_)^2^)	3	[−1, 2]	−3.86
*F*_20_(*x*)=−∑_*i*=1_^4^*c*_*i*_exp(−∑_*j*=1_^6^*a*_*ij*_(*x*_*j*_ − *p*_*ij*_)^2^)	6	[0, 1]	−3.32
*F*_21_(*x*)=−∑_*i*=1_^5^[(*X* − *a*_*i*_)(*X* − *a*_*i*_)^*T*^+*c*_*i*_]^−1^	4	[0, 10]	−10.1532
*F*_22_(*x*)=−∑_*i*=1_^7^[(*X* − *a*_*i*_)(*X* − *a*_*i*_)^*T*^+*c*_*i*_]^−1^	4	[0, 10]	−10.4028
*F*_23_(*x*)=−∑_*i*=1_^10^[(*X* − *a*_*i*_)(*X* − *a*_*i*_)^*T*^+*c*_*i*_]^−1^	4	[0, 10]	−10.5363

**Table 4:** Results of algorithms on 23 standard benchmark functions.

Function		RLTLBO	TLBO	mGWO	MALO	DSCA	HOA	AO	HHO	SSA
F1	Mean	0.00*E + *00	3.90*E − *79	4.26*E − *19	1.37*E − *03	2.55*E − *288	3.13*E − *136	2.34*E − *104	8.97*E − *98	1.30*E − *07
	Std	0.00*E + *00	6.59*E − *79	1.08*E − *18	1.56*E − *03	0.00*E + *00	1.21*E − *135	1.08*E − *103	4.16*E − *97	1.09*E − *07
F2	Mean	1.29*E − *223	4.17*E − *40	3.37*E − *12	6.86*E + *01	5.92*E − *171	4.44*E − *68	2.82*E − *53	1.34*E − *48	1.79*E + *00
	Std	0.00*E + *00	3.21*E − *40	2.54*E − *12	4.90*E + *01	0.00*E + *00	2.42*E − *67	1.13*E − *52	5.75*E − *48	1.15*E + *00
F3	Mean	0.00*E + *00	2.50*E − *17	6.41*E − *01	4.81*E + *03	1.43*E − *241	2.23*E + *02	2.22*E − *101	7.16*E − *79	1.61*E + *03
	Std	0.00*E + *00	4.35*E − *17	1.46*E + *00	2.18*E + *03	0.00*E + *00	5.03*E + *02	1.22*E − *100	3.56*E − *78	1.03*E + *03
F4	Mean	3.07*E − *221	1.72*E − *32	2.42*E − *03	1.64*E + *01	1.97*E − *134	5.04*E − *65	3.20*E − *53	2.51*E − *48	1.11*E + *01
	Std	0.00*E + *00	1.76*E − *32	3.02*E − *03	4.23*E + *00	1.08*E − *133	1.84*E − *64	1.75*E − *52	8.46*E − *48	3.74*E + *00
F5	Mean	2.65*E + *01	2.42*E + *01	2.64*E + *01	9.86*E − *01	2.85*E + *01	2.89*E + *01	6.82*E − *03	1.22*E − *02	2.55*E + *02
	Std	4.01*E − *01	7.41*E − *01	8.44*E − *01	5.21*E + *00	3.59*E − *01	7.45*E − *02	1.66*E − *02	1.79*E − *02	3.44*E + *02
F6	Mean	9.03*E − *02	2.57*E − *06	4.54*E − *01	5.00*E − *04	6.01*E + *00	6.46*E + *00	4.43*E − *05	9.58*E − *05	1.28*E − *07
	Std	1.15*E − *01	7.98*E − *06	3.20*E − *01	3.05*E − *04	1.61*E − *01	4.76*E − *01	6.15*E − *05	1.24*E − *04	1.13*E − *07
F7	Mean	3.57*E − *05	1.12*E − *03	4.61*E − *03	1.05*E − *04	2.54*E − *04	5.88*E − *02	9.62*E − *05	1.68*E − *04	1.81*E − *01
	Std	4.71*E − *05	3.06*E − *04	1.64*E − *03	7.89*E − *05	2.88*E − *04	4.10*E − *02	7.92*E − *05	1.36*E − *04	8.96*E − *02
F8	Mean	−7.36*E + *03	−7.85*E + *03	−6.58*E + *03	−1.22*E + *04	−3.96*E + *03	−4.30*E + *03	−8.92*E + *03	−1.25*E + *04	−7.56*E + *03
	Std	6.78*E + *02	9.32*E + *02	1.24*E + *03	1.08*E + *03	4.31*E + *02	7.82*E + *02	3.77*E + *03	8.42*E + *01	7.07*E + *02
F9	Mean	0.00*E + *00	1.41*E + *01	1.70*E + *01	8.44*E + *01	0.00*E + *00	5.06*E + *01	0.00*E + *00	0.00*E + *00	5.19*E + *01
	Std	0.00*E + *00	6.20*E + *00	9.11*E + *00	3.15*E + *01	0.00*E + *00	9.32*E + *01	0.00*E + *00	0.00*E + *00	1.88*E + *01
F10	Mean	8.88*E − *16	7.05*E − *15	1.14*E + *00	4.77*E + *00	8.88*E − *16	6.10*E − *15	8.88*E − *16	8.88*E − *16	2.62*E + *00
	Std	0.00*E + *00	1.60*E − *15	1.88*E + *00	2.64*E + *00	0.00*E + *00	2.42*E − *15	0.00*E + *00	0.00*E + *00	8.98*E − *01
F11	Mean	0.00*E + *00	3.29*E − *04	4.86*E − *03	6.05*E − *02	0.00*E + *00	1.18*E − *01	0.00*E + *00	0.00*E + *00	2.24*E − *02
	Std	0.00*E + *00	1.80*E − *03	9.13*E − *03	2.33*E − *02	0.00*E + *00	2.57*E − *01	0.00*E + *00	0.00*E + *00	1.45*E − *02
F12	Mean	8.32*E − *04	5.38*E − *07	3.51*E − *02	1.60*E − *05	8.37*E − *01	1.23*E + *00	3.04*E − *06	1.02*E − *05	7.22*E + *00
	Std	1.52*E − *03	2.76*E − *06	4.56*E − *02	1.16*E − *05	1.08*E − *01	2.42*E − *01	4.59*E − *06	1.12*E − *05	3.01*E + *00
F13	Mean	2.00*E + *00	7.41*E − *02	3.83*E − *01	1.70*E − *03	2.76*E + *00	3.08*E + *00	4.57*E − *05	8.69*E − *05	2.19*E + *01
	Std	1.17*E + *00	8.70*E − *02	2.15*E − *01	3.95*E − *03	5.11*E − *02	1.83*E − *01	1.18*E − *04	9.70*E − *05	1.44*E + *01
F14	Mean	1.06*E + *00	9.98*E − *01	9.98*E − *01	1.46*E + *00	1.35*E + *00	2.78*E + *00	4.06*E + *00	1.36*E + *00	1.16*E + *00
	Std	3.62*E − *01	0.00*E + *00	3.81*E − *12	7.69*E − *01	6.1*E − *01	2.07*E + *00	4.46*E + *00	9.52*E − *01	4.57*E − *01
F15	Mean	3.55*E − *04	3.82*E − *04	3.04*E − *03	1.40*E − *03	8.91*E − *04	6.77*E − *03	5.00*E − *04	4.01*E − *04	3.55*E − *03
	Std	1.02*E − *04	1.54*E − *04	6.91*E − *03	3.62*E − *03	3.99*E − *04	5.47*E − *03	1.10*E − *04	2.36*E − *04	6.71*E − *03
F16	Mean	−1.03*E + *00	−1.03*E + *00	−1.03*E + *00	−1.03*E + *00	−1.03*E + *00	−9.99*E − *01	−1.03*E + *00	−1.03*E + *00	−1.03*E + *00
	Std	6.58*E − *16	6.95*E − *16	3.39*E − *08	1.65*E − *13	3.99*E − *04	3.29*E − *02	3.01*E − *04	3.76*E − *09	1.83*E − *14
F17	Mean	3.98*E − *01	3.98*E − *01	3.98*E − *01	3.98*E − *01	4.09*E − *01	3.99*E − *01	3.98*E − *01	3.98*E − *01	3.98*E − *01
	Std	0.00*E + *00	0.00*E + *00	6.52*E − *09	5.57*E − *14	1.06*E − *02	1.08*E − *03	1.09*E − *04	4.60*E − *06	7.21*E − *15
F18	Mean	3.00*E + *00	3.00*E + *00	3.00*E + *00	3.00*E + *00	3.00*E + *00	4.94*E + *00	3.03*E + *00	3.00*E + *00	3.00*E + *00
	Std	4.95*E − *16	1.24*E − *15	1.03*E − *07	5.76*E − *13	8.33*E − *04	6.82*E + *00	5.73*E − *02	3.88*E − *07	2.87*E − *13
F19	Mean	−3.86*E + *00	−3.86*E + *00	−3.86*E + *00	−3.86*E + *00	−3.82*E + *00	−3.86*E + *00	−3.85*E + *00	−3.86*E + *00	−3.86*E + *00
	Std	2.71*E − *15	3.16*E − *15	1.08*E − *06	6.39*E − *13	2.33*E − *02	6.99*E − *04	6.96*E − *03	2.07*E − *03	1.09*E − *12
F20	Mean	−3.31*E + *00	−3.30*E + *00	−3.23*E + *00	−3.23*E + *00	−2.80*E + *00	−3.25*E + *00	−3.16*E + *00	−3.08*E + *00	−3.23*E + *00
	Std	2.95*E − *02	4.12*E − *02	6.47*E − *02	5.14*E − *02	2.71*E − *01	9.05*E − *02	8.91*E − *02	1.22*E − *01	6.22*E − *02
F21	Mean	−1.02*E + *01	−1.02*E + *01	−9.98*E + *00	−7.62*E + *00	−3.27*E + *00	−9.43*E + *00	−1.01*E + *01	−5.18*E + *00	−8.07*E + *00
	Std	6.04*E − *09	1.41*E − *03	9.30*E − *01	2.82*E + *00	1.54*E + *00	9.62*E − *01	2.09*E − *02	7.51*E − *01	3.28*E + *00
F22	Mean	−1.04*E + *01	−1.01*E + *01	−1.04*E + *01	−7.06*E + *00	−3.87*E + *00	−9.36*E + *00	−1.04*E + *01	−5.08*E + *00	−9.32*E + *00
	Std	1.23*E − *07	1.25*E + *00	4.45*E − *04	3.48*E + *00	1.17*E + *00	1.69*E + *00	5.50*E − *02	6.94*E − *03	2.51*E + *00
F23	Mean	−1.05*E + *01	−1.01*E + *01	−1.05*E + *01	−7.31*E + *00	−4.19*E + *00	−9.63*E + *00	−1.05*E + *01	−5.24*E + *00	−7.89*E + *00
	Std	1.57*E − *07	1.57*E + *00	3.42*E − *04	3.55*E + *00	1.11*E + *00	1.52*E + *00	2.23*E − *02	9.58*E − *01	3.59*E + *00

**Table 5:** *p*-Values from the Wilcoxon rank-sum test for the results in Table 4.

Function	RLTLBO vs.							
	TLBO	mGWO	MALO	DSCA	HOA	AO	HHO	SSA
F1	6.10*E − *05	6.10*E − *05	6.10*E − *05	NaN	6.10*E − *05	6.10*E − *05	6.10*E − *05	6.10*E − *05
F2	6.10*E − *05	6.10*E − *05	6.10*E − *05	6.10*E − *04	6.10*E − *05	6.10*E − *05	6.10*E − *05	6.10*E − *05
F3	6.10*E − *05	6.10*E − *05	6.10*E − *05	1.56*E − *02	6.10*E − *05	6.10*E − *05	6.10*E − *05	6.10*E − *05
F4	6.10*E − *05	6.10*E − *05	6.10*E − *05	6.10*E − *05	6.10*E − *05	6.10*E − *05	6.10*E − *05	6.10*E − *05
F5	6.10*E − *05	3.30*E − *01	6.10*E − *05	6.10*E − *05	6.10*E − *05	6.10*E − *05	6.10*E − *05	8.54*E − *04
F6	6.10*E − *05	1.22*E − *04	6.10*E − *05	6.10*E − *05	6.10*E − *05	6.10*E − *05	6.10*E − *05	6.10*E − *05
F7	6.10*E − *05	6.10*E − *05	4.89*E − *01	6.10*E − *04	6.10*E − *05	4.89*E − *01	7.30*E − *02	6.10*E − *05
F8	0.010254	6.37*E − *02	6.10*E − *05	6.10*E − *05	6.10*E − *05	1.21*E − *01	6.10*E − *05	5.61*E − *01
F9	6.10*E − *05	6.10*E − *05	6.10*E − *05	NaN	1.25*E − *01	NaN	NaN	6.10*E − *05
F10	6.10*E − *05	6.10*E − *05	6.10*E − *05	NaN	6.10*E − *05	NaN	NaN	6.10*E − *05
F11	NaN	1.95*E − *03	6.10*E − *05	NaN	3.12*E − *02	NaN	NaN	6.10*E − *05
F12	6.10*E − *05	6.10*E − *05	6.10*E − *05	6.10*E − *05	6.10*E − *05	6.10*E − *05	6.10*E − *05	6.10*E − *05
F13	3.05*E − *04	6.10*E − *04	6.10*E − *05	3.89*E − *01	2.01*E − *03	6.10*E − *05	6.10*E − *05	3.05*E − *04
F14	NaN	6.10*E − *05	6.10*E − *05	6.10*E − *05	6.10*E − *05	6.10*E − *05	6.10*E − *05	6.10*E − *05
F15	8.90*E − *01	2.01*E − *03	1.83*E − *04	6.10*E − *05	6.10*E − *05	6.10*E − *05	8.36*E − *03	6.10*E − *05
F16	NaN	6.10*E − *05	6.10*E − *05	6.10*E − *05	6.10*E − *05	6.10*E − *05	1.22*E − *04	6.10*E − *05
F17	NaN	6.10*E − *05	2.44*E − *04	6.10*E − *05	6.10*E − *05	6.10*E − *05	6.10*E − *05	9.76*E − *04
F18	NaN	6.10*E − *05	6.10*E − *05	6.10*E − *05	6.10*E − *05	6.10*E − *05	6.10*E − *05	6.10*E − *05
F19	NaN	6.10*E − *05	6.10*E − *05	6.10*E − *05	6.10*E − *05	6.10*E − *05	6.10*E − *05	6.10*E − *05
F20	8.52*E − *01	4.13*E − *02	1.35*E − *01	6.10*E − *05	2.01*E − *03	6.10*E − *05	6.10*E − *05	3.05*E − *04
F21	1.68*E − *01	6.10*E − *05	4.79*E − *02	6.10*E − *05	6.10*E − *05	6.10*E − *05	6.10*E − *05	1.03*E − *02
F22	6.25*E − *02	6.10*E − *05	2.56*E − *02	6.10*E − *05	6.10*E − *05	6.10*E − *05	6.10*E − *05	4.13*E − *02
F23	7.81*E − *03	6.10*E − *05	6.10*E − *05	6.10*E − *05	6.10*E − *05	6.10*E − *05	6.10*E − *05	2.56*E − *02

**Table 6:** Descriptions of the benchmark functions from CEC2017.

Function	Name	Dim	Range	*f*_min_
Hybrid functions (N is basic number of functions)				
C13	Hybrid function 3 (*N* = 3)	10	[−100, 100]	1300
C14	Hybrid function 4 (*N* = 4)	10	[−100, 100]	1400
C15	Hybrid function 5 (*N* = 4)	10	[−100, 100]	1500
C19	Hybrid function 6 (*N* = 5)	10	[−100, 100]	1900
Composite functions (N is basic number of functions)				
C22	Composite function 2 (*N* = 3)	10	[−100, 100]	2200
C25	Composite function 5 (*N* = 5)	10	[−100, 100]	2500
C28	Composite function 8 (*N* = 6)	10	[−100, 100]	2800
C29	Composite function 9 (*N* = 6)	10	[−100, 100]	2900

**Table 7:** Comparison results of algorithms on CEC2017.

Function		RLTLBO	TLBO	mGWO	MALO	DSCA	HOA	AO	HHO	SSA
C13	Mean	4.38*E + *03	6.04*E + *03	4.35*E + *03	1.78*E + *04	6.25*E + *05	1.53*E + *06	1.77*E + *04	1.70*E + *04	1.46*E + *04
	Std	2.76*E + *03	4.33*E + *03	2.99*E + *03	1.30*E + *04	4.55*E + *05	1.28*E + *06	1.39*E + *04	1.03*E + *04	1.29*E + *04
C14	Mean	1.46*E + *03	1.47*E + *03	1.47*E + *03	2.75*E + *03	4.78*E + *03	3.87*E + *03	2.36*E + *03	2.20*E + *03	3.35*E + *03
	Std	1.81*E + *01	2.40*E + *01	1.98*E + *01	2.02*E + *03	3.76*E + *03	1.99*E + *03	1.12*E + *03	1.05*E + *03	3.10*E + *03
C15	Mean	1.62*E + *03	1.73*E + *03	1.74*E + *03	8.28*E + *03	7.97*E + *03	2.49*E + *04	5.91*E + *03	7.35*E + *03	1.06*E + *04
	Std	5.96*E + *01	1.44*E + *02	2.36*E + *02	5.72*E + *03	3.62*E + *03	1.54*E + *04	2.16*E + *03	3.10*E + *03	7.51*E + *03
C19	Mean	2.00*E + *03	2.11*E + *03	2.65*E + *03	1.54*E + *04	3.37*E + *04	1.69*E + *04	2.10*E + *04	1.67*E + *04	8.46*E + *03
	Std	9.63*E + *00	3.19*E + *02	1.68*E + *03	1.23*E + *04	3.00*E + *04	1.34*E + *04	2.88*E + *04	1.37*E + *04	6.44*E + *03
C22	Mean	2.30*E + *03	2.30*E + *03	2.30*E + *00	2.30*E + *03	2.55*E + *03	2.47*E + *03	2.31*E + *03	2.41*E + *03	2.33*E + *03
	Std	1.99*E + *01	8.68*E + *00	9.25*E − *01	2.88*E + *01	8.10*E + *01	4.58*E + *02	5.85*E + *00	3.85*E + *02	1.69*E + *02
C25	Mean	2.92*E + *03	2.93*E + *03	2.92*E + *03	2.93*E + *03	3.12*E + *03	2.97*E + *03	2.94*E + *03	2.93*E + *03	2.92*E + *03
	Std	2.32*E + *01	2.41*E + *01	2.33*E + *01	2.38*E + *01	6.48*E + *01	2.35*E + *01	2.50*E + *01	6.24*E + *01	2.45*E + *01
C28	Mean	3.23*E + *03	3.30*E + *03	3.33*E + *03	3.31*E + *03	3.40*E + *03	3.50*E + *03	3.44*E + *03	3.45*E + *03	3.29*E + *03
	Std	1.15*E + *02	1.60*E + *02	1.12*E + *02	1.47*E + *02	9.48*E + *01	1.06*E + *02	1.09*E + *02	1.45*E + *02	1.68*E + *02
C29	Mean	3.18*E + *03	3.19*E + *03	3.17*E + *03	3.27*E + *03	3.38*E + *03	3.38*E + *03	3.26*E + *03	3.37*E + *03	3.27*E + *03
	Std	1.84*E + *01	2.16*E + *01	2.13*E + *01	6.15*E + *01	5.77*E + *01	6.58*E + *01	5.87*E + *01	1.20*E + *02	7.20*E + *01

**Table 8:** *p* values from the Wilcoxon rank-sum test for the results in Table 7.

Function	RLTLBO vs.							
	TLBO	mGWO	MALO	DSCA	HOA	AO	HHO	SSA
C13	2.90*E − *02	3.59*E − *01	1.81*E − *02	6.10*E − *05	6.10*E − *05	6.10*E − *05	3.36*E − *03	1.22*E − *04
C14	1.35*E − *01	4.37*E − *02	6.10*E − *05	6.10*E − *05	6.10*E − *05	6.10*E − *05	1.83*E − *04	6.10*E − *05
C15	3.36*E − *03	8.36*E − *03	6.10*E − *05	6.10*E − *05	6.10*E − *05	6.10*E − *05	6.10*E − *05	6.10*E − *05
C19	1.24*E − *02	3.30*E − *02	8.54*E − *04	6.10*E − *05	6.10*E − *05	6.10*E − *05	6.10*E − *05	6.10*E − *05
C22	4.27*E − *03	4.13*E − *02	4.04*E − *02	6.10*E − *05	6.10*E − *05	6.10*E − *05	6.10*E − *05	8.47*E − *02
C25	5.61*E − *01	8.47*E − *01	8.47*E − *01	6.10*E − *05	2.01*E − *03	1.21*E − *02	1.69*E − *02	3.62*E − *01
C28	2.48*E − *02	1.51*E − *02	4.79*E − *02	1.81*E − *02	1.53*E − *03	6.10*E − *04	5.37*E − *03	4.21*E − *02
C29	4.54*E − *03	6.10*E − *04	6.10*E − *05	6.10*E − *05	6.10*E − *05	8.54*E − *04	6.10*E − *05	1.51*E − *02

**Table 9:** Comparison results for the welded beam design problem.

Algorithm	Optimum variables				Optimum cost
	*h*	*l*	*t*	*b*	
RLTLBO	0.205730	3.253000	9.036600	0.205730	1.695200
SMA [50]	0.205400	3.258900	9.038400	0.205800	1.696040
WOA [14]	0.205396	3.484293	9.037426	0.206276	1.730499
MPA [51]	0.205728	3.470509	9.036624	0.205730	1.724853
MVO [52]	0.205463	3.473193	9.044502	0.205695	1.726450
GA [6]	0.248900	6.173000	8.178900	0.253300	2.430000
HS [53]	0.244200	6.223100	8.291500	0.240000	2.380700

**Table 10:** Comparison results for the pressure vessel design problem.

Algorithm	Optimum variables				Optimum cost
	Ts	Th	*R*	*L*	
RLTLBO	0.7698901	0.4201098	42.536830	171.348900	5926.77920
AO [15]	1.0540000	0.1828060	59.621900	38.8050000	5949.22580
SMA [50]	0.7931000	0.3932000	40.671100	196.217800	5994.18570
WOA [14]	0.8125000	0.4375000	42.098270	176.638998	6059.74100
GWO [13]	0.8125000	0.4345000	42.089200	176.758700	6051.56390
MVO [52]	0.8125000	0.4375000	42.090738	176.738690	6060.80660
GA [6]	0.8125000	0.4375000	42.097398	176.654050	6059.94634
ES [54]	0.8125000	0.4375000	42.098087	176.640518	6059.74560

**Table 11:** Comparison results for the tension/compression spring design problem.

Algorithm	Optimum variables			Optimum weight
	*d*	*D*	*N*	
RLTLBO	0.0551180	0.505900	5.1167000	0.01093800
AO [15]	0.0502439	0.352620	10.542500	0.01116500
SSA [12]	0.0512070	0.345215	12.004032	0.01267630
WOA [14]	0.0512070	0.345215	12.004032	0.01267630
GWO [13]	0.0516900	0.356737	11.288850	0.01266600
PSO [11]	0.0517280	0.357644	11.244543	0.01267470
GA [6]	0.0514800	0.351661	11.632201	0.01270478
HS [53]	0.0511540	0.349871	12.076432	0.01267060

**Table 12:** Comparison results for the speed reducer design problem.

Algorithm	Optimum variables							Optimum weight
	x1	x2	x3	x4	x5	x6	x7	
RLTLBO	3.497600	0.7000	17.0000	7.30000	7.800000	3.350060	5.285530	2995.43740
AO [15]	3.502100	0.7000	17.0000	7.30990	7.747600	3.364100	5.299400	3007.73280
PSO [11]	3.500100	0.7000	17.0002	7.51770	7.783200	3.350800	5.286700	3145.92200
AOA [9]	3.503840	0.7000	17.0000	7.30000	7.729330	3.356490	5.286700	2997.91570
GA [6]	3.510253	0.7000	17.0000	8.35000	7.800000	3.362201	5.287723	3067.56100
SCA [55]	3.508755	0.7000	17.0000	7.30000	7.800000	3.461020	5.289213	3030.56300
HS [53]	3.520124	0.7000	17.0000	8.37000	7.800000	3.366970	5.288719	3029.00200
FA [56]	3.507495	0.7001	17.0000	7.719674	8.080854	3.351512	5.287051	3010.13749

**Table 13:** Comparison results for the thre*E − *bar truss design problem.

Algorithm	Optimum variables		Optimum weight
	x1	x2	
RLTLBO	0.788420000000000	0.408110000000000	263.852300000000
AO [15]	0.792600000000000	0.396600000000000	263.868400000000
SSA [12]	0.788665410000000	0.408275784000000	263.895840000000
AOA [9]	0.793690000000000	0.394260000000000	263.915400000000
MVO [52]	0.788602760000000	0.408453070000000	263.895849900000
GOA [57]	0.788897555578973	0.407619570115153	263.895881496069

**Table 14:** Comparison results for the car crashworthiness design problem.

Algorithm	RLTLBO	DE [7]	GA [6]	FA [55]	CS [59]	GOA [57]	EOBL-GOA [58]
x1	0.50000	0.50000	0.50005	0.50000	0.50000	0.50000	0.50000
x2	1.11621	1.11670	1.28017	1.36000	1.11643	1.11670	1.11643
x3	0.50000	0.50000	0.50001	0.50000	0.50000	0.50000	0.50000
x4	1.30215	1.30208	1.03302	1.20200	1.30208	1.30208	1.30208
x5	0.50000	0.50000	0.50001	0.50000	0.50000	0.50000	0.50000
x6	1.50000	1.50000	0.50000	1.12000	1.50000	1.50000	1.50000
x7	0.50000	0.50000	0.50000	0.50000	0.50000	0.50000	0.50000
x8	0.34500	0.34500	0.34994	0.34500	0.34500	0.34500	0.34500
x9	0.332814	0.192000	0.192000	0.192000	0.192000	0.192000	0.192000
x10	−19.58840	−19.54935	10.31190	8.87307	−19.54935	−19.54935	−19.54935
x11	0.019066	−0.004310	0.001670	−18.998080	−0.004310	−0.004310	−0.004310
Optimal weight	22.84240	22.84298	22.85653	22.84298	22.84294	22.84474	22.84294

**Table 15:** Comparison results for the tubular column design problem.

Algorithm	Optimum variables		Optimum cost
	*d*	*t*	
RLTLBO	5.45120	0.29196	26.53130
mGWO	5.45080	0.29201	26.53270
DSCA	5.50250	0.29214	26.79030
HOA	5.26260	0.35487	28.86470
AO	5.46300	0.29656	26.83540
HHO	5.44380	0.29313	26.55820
CS [59]	5.45139	0.29196	26.53217

**Table 16:** Comparison results for the frequency-modulated sound waves design problem.

Algorithm	Optimum variables						Optimum cost
	a1	*ω*1	a2	*ω*2	a3	*ω*3	
RLTLBO	−0.97498	−5.0327	−1.5640	−4.7840	−2.0060	4.9055	0.21738
GWO [13]	−0.66540	−0.1684	1.5173	−0.1287	−4.1335	−4.8997	8.47250
MFO [61]	0.61410	0.0432	−4.3251	4.7923	0.8339	0.1278	11.89690
PSO [11]	−0.58860	5.0145	−3.2779	−4.9324	−0.8562	−0.1476	13.18070
TSA [62]	0.34150	4.7881	1.4309	0.1158	0.0975	0.5480	25.10520
FFA [63]	−0.56270	0.0525	−3.4797	4.8930	1.1491	−4.8345	17.42910

## Data Availability

The data used to support the findings of this study are available from the corresponding author upon request.
